# Forensic DNA Recovery from FFPE Tissue Using the Maxwell^®^ RSC Xcelerate Kit: Optimization, Challenges, and Limitations

**DOI:** 10.3390/genes16091074

**Published:** 2025-09-12

**Authors:** Dagmara Lisman, Andrzej Ossowski, Aleksandra Tołoczko-Grabarek, Mateusz Kozłowski, Aneta Cymbaluk-Płoska

**Affiliations:** 1 Department of Genomic and Forensic Genetics, Pomeranian Medical University in Szczecin, 70-111 Szczecin, Poland; andrzej.ossowski@pum.edu.pl; 2 Department of Pathomorphology, Pomeranian Medical University in Szczecin, University Clinical Hospital No. 2 in Szczecin, 70-111 Szczecin, Poland; 3 Department of Reconstructive and Oncological Gynecology, Pomeranian Medical University in Szczecin, 70-111 Szczecin, Poland; mateusz.kozlowski@pum.edu.pl (M.K.); aneta.cymbaluk.ploska@pum.edu.pl (A.C.-P.)

**Keywords:** formalin-fixed paraffin-embedded (FFPE), DNA degradation, short tandem repeats (STR), forensic genetics, DNA extraction

## Abstract

**Background/Objectives:** Obtaining reliable DNA profiles from archival tissue preserved as formalin-fixed, paraffin-embedded (FFPE) samples remains a major challenge in both forensic and medical evaluations. The quality of DNA isolated from FFPE material is frequently compromised due to formalin-induced fragmentation and chemical modifications. These limitations are particularly relevant in cases of suspected medical malpractice related to cancer diagnosis or treatment, where retrospective molecular analyses may provide critical evidence. The aim of this study was to evaluate the performance of the Maxwell^®^ RSC Xcelerate DNA FFPE Kit (Promega) in generating DNA profiles from archival FFPE tissue blocks of endometrial cancer and to identify the limitations associated with this approach. **Methods:** Archival FFPE blocks of endometrial cancer were analyzed using the Maxwell^®^ RSC Xcelerate DNA FFPE Kit. DNA yield, purity, and degradation indices were assessed using standard real-time PCR-based quantification methods. Short tandem repeat (STR) profiling was performed with forensic genotyping kits, and the completeness, allele balance, and reliability of obtained profiles were evaluated. The obtained results were compared with reference quality thresholds commonly used in forensic practice. **Results:** The Maxwell^®^ RSC Xcelerate Kit allowed for recovery of relatively high DNA yields with consistently low degradation indices, confirming good extraction efficiency from FFPE samples. Nevertheless, despite favorable quantitative values, the generation of complete STR profiles was often unsuccessful. Partial or incomplete profiles were frequent, characterized by allele dropout and imbalance, which substantially reduced their evidentiary value. These findings suggest that DNA fragmentation and fixation-related artifacts impair amplification efficiency and limit the usefulness of STR analysis. **Conclusions:** This study emphasizes the persistent challenges of DNA profiling from FFPE tissue in forensic-medical contexts. Although the Maxwell^®^ RSC Xcelerate Kit demonstrated effective DNA recovery, the ability to generate complete and interpretable STR profiles remained limited. Further refinement of extraction protocols, as well as improved interpretative strategies, are required to enhance the reliability and evidentiary significance of molecular analyses based on archival FFPE material.

## 1. Introduction

Embedding tissues in paraffin (FFPE—Formalin-Fixed, Paraffin-Embedded) is the standard method for preparing histopathological samples, particularly in cancer diagnostics. One of the main objectives of this process is to preserve the tissue architecture and cellular morphology [1]. In routine histopathological procedures, the most commonly used fixative is 10% neutral-buffered formalin (NBF), which corresponds to a 4% aqueous solution of formaldehyde. Buffering, most often phosphate-based, ensures a neutral pH of around 7.0 [2]. In specialized studies, alternative variants of formalin may also be used, for example: 4% formaldehyde in PBS for immunohistochemistry, methacarn (“modified” formalin) for better preservation of lipid structures, or 4% paraformaldehyde (PFA) for molecular biology and electron microscopy. The choice of the fixative depends on the intended downstream application of the biological material [3].

Another reagent widely used in histopathological studies is paraffin. It stabilizes tissue structure, prevents morphological degradation, and enables long-term storage. Paraffin also protects tissues from mechanical damage—its hardened structure allows for precise sectioning of thin slices. Additionally, it minimizes structural alterations, ensuring that well-preserved tissues retain their cellular organization and histological details. An important aspect of paraffin embedding is the replacement of water and lipids—during embedding, tissues are dehydrated and infiltrated with paraffin, which stabilizes their structure. However, paraffin may also exert negative effects on tissues. It can cause cytoplasmic condensation, altering cellular appearance, and may contribute to DNA and RNA fragmentation, which complicates molecular analyses such as PCR or sequencing [4,5,6].

Formalin, on the other hand, preserves tissue by creating cross-links between proteins, preventing autolysis (self-destruction of cells) and tissue degradation. Paraffin complements this process by stabilizing tissue architecture, thereby allowing precise sectioning of ultrathin slices with a microtome for microscopic analysis. FFPE tissues are the gold standard in cancer diagnostics, enabling both classification and assessment of tumor aggressiveness [3,7,8]. Such preserved tissues can be stored for many years, allowing their re-analysis if needed, for example, in the context of additional genetic or immunohistochemical tests. This long-term stability is also crucial for retrospective studies, biomarker discovery, and clinical research [8,9].

The process of tissue fixation induces a series of chemical changes that affect DNA quality and integrity. Formalin, a solution of formaldehyde, forms cross-linking bonds between proteins and nucleic acids, which hinders DNA extraction and amplification [3]. The formation of methylene bridges between nitrogenous bases results in DNA fragmentation, leading to difficulties in obtaining complete genetic profiles. Moreover, prolonged storage in formalin enhances hydrolytic processes, causing further degradation of genetic material [10].

After fixation in formalin, tissue samples are embedded in paraffin, which protects the specimen from external factors but at the same time may hinder efficient DNA recovery [11]. Incomplete removal of formalin before paraffin embedding can result in further DNA degradation and fragmentation. The paraffinization process may also stabilize unwanted chemical bonds, which additionally complicates DNA extraction and reduces the efficiency of PCR amplification. As a result, FFPE material is often characterized by low amplification yield and an increased occurrence of artifacts in genetic analyses [12].

In forensic medicine and forensic genetics, FFPE tissues are used for identification analyses in situations where they constitute the only available biological material in criminal cases from many years ago, enabling examination even when other DNA sources have undergone degradation [13]. Post-mortem diagnostics also allow for the detection of mutations associated with hereditary diseases, cancers, or other conditions that may have contributed to the cause of death. Their analysis can play a crucial role in cases involving medical malpractice, particularly when there is a need for a retrospective evaluation of the accuracy of a diagnosis, the applied treatment, or clinical decisions. This is especially important in assessing whether errors occurred during the preparation of histopathological samples, such as patient sample mix-ups or material contamination [14,15].

DNA can be recovered from formalin-fixed, paraffin-embedded (FFPE) tissues; however, the process is considerably more challenging than with fresh or frozen samples. Formalin induces DNA fragmentation and creates cross-links between proteins and nucleic acids, which can hinder both extraction and amplification. It leads to the formation of stable bonds between DNA and proteins, thereby reducing extraction efficiency [6,16]. In some cases, the amount of sample available may be small or insufficient to perform a complete analysis, requiring the use of optimized protocols, specialized extraction kits, and experienced laboratory personnel. FFPE preparations may also contain residual formalin, which introduces sequencing errors by generating mutational artifacts [17,18]. The short length of DNA fragments further limits the effectiveness of analytical methods, particularly those that rely on long amplicons, such as short tandem repeat (STR) analysis, commonly applied in forensic genetics. DNA quality in FFPE tissues is also strongly influenced by storage time and conditions; samples stored for many years often show additional degradation, which further complicates analysis [12,19].

Due to DNA degradation and potential histological artifacts, it is necessary to employ advanced analytical methods, such as molecular and immunohistochemical techniques [5]. A comprehensive evaluation requires an interdisciplinary approach, involving collaboration among pathologists, geneticists, and forensic experts. To minimize the inherent limitations of FFPE samples, various strategies have been developed to improve the quality of extracted DNA. One such strategy is the optimization of extraction procedures, including the use of protein-degrading enzymes (e.g., proteinase K) and modified DNA isolation protocols. Another is the use of amplification methods targeting small DNA fragments—such as short amplicon markers (miniSTRs)—which perform better with degraded DNA [5,20]. Next-generation sequencing (NGS) technologies also enable the analysis of highly degraded samples by employing specialized error-correction algorithms. Additionally, careful deparaffinization and limiting excessive exposure to formalin during sample preparation can improve DNA yield and quality [21].

There is a common misconception that the preparation of FFPE tissue completely destroys the DNA it contains. Nothing could be further from the truth. Although the DNA is certainly damaged and fragmented, it is not entirely lost. Formalin itself leads to the formation of methyl bridges and DNA crosslinking, which hinder subsequent isolation and analysis [22,23]. Prolonged fixation, combined with exposure to high temperatures during paraffin embedding, often results in chemical modifications of DNA, such as cytosine deamination to uracil, which may introduce sequencing errors [18,24]. This process also causes extensive crosslinking with proteins, further complicating the recovery of “pure” DNA.

Despite these limitations, FFPE tissues remain a viable source of DNA for identification studies, although the quality of the material is inherently restricted [13]. These protocols often include steps to reverse protein–DNA crosslinks while simultaneously shortening the extraction process. Enzymatic DNA repair, for example, through enzymes capable of reversing chemical modifications, can further enhance the quality of recovered DNA [18,20,25].

Another critical factor is avoiding excessively long fixation times in formalin (>24–48 h), which markedly increases DNA damage. The use of buffered formalin instead of unbuffered formalin also has a significant impact on DNA quality. Regular formalin is acidic (pH < 4), leading to intense DNA degradation and higher rates of mutations, including cytosine-to-uracil deamination. Buffered formalin (commonly phosphate-buffered, pH ~ 7) stabilizes the environment, limiting hydrolysis and DNA fragmentation. Acidic conditions promote strong DNA–protein crosslinking, hindering subsequent extraction and increasing spontaneous DNA mutations (e.g., C > T transitions), which may interfere with genetic analyses. Buffered formalin reduces this process, allowing for the recovery of longer DNA fragments. DNA isolated from tissues fixed in buffered formalin may reach lengths of up to ~1 kb, compared to only 100–300 bp typically observed with unbuffered formalin. This substantially improves the usability of the material in molecular biology applications [3,18,26].

We have also observed these differences in our own molecular analyses. Material obtained from small regional hospitals, where unbuffered formalin was used for tissue fixation, consistently yielded inferior DNA results.

## 2. Material and Methods

The material used for the study consisted of FFPE tissues obtained from 25 patients diagnosed with advanced endometrial cancer. The material was collected during 2023/24. The study received approval from the Bioethics Committee of the Pomeranian Medical University (KB-006/49/2022 and KB/006/74/2024).

The studies are part of a project funded under the National Science Centre competition (grant number: 2024/08/X/NZ1/00695). All patients provided informed consent to participate in the study and for the scientific investigations resulting from this work.

The paraffin blocks were stored at room temperature for at least 30 min prior to sectioning (blocks that are too cold become brittle, while those that are too warm become soft). In cases of uneven block surfaces or oxidation, preliminary trimming was performed. The block was mounted on a microtome, ensuring that the cutting surface was perpendicular to the blade. Sections of 10 µm thickness were cut, as this is the standard thickness used for DNA analysis. Ten sections were placed into a sterile Eppendorf tube and stored at 4–8 °C until DNA isolation.

Archival FFPE blocks of endometrial cancer were obtained from the pathology archives. For each block, the exact archival time (years since fixation and embedding) was recorded in order to evaluate the potential effect of storage duration on DNA quality.

### 2.1. DNA Extraction

For all tumor samples, a minimum of 20% tumor cell content was required. Prior to DNA extraction, the percentage of tumor cells was assessed independently by two pathologists. The proportion of tumor cells was defined as the ratio of tumor cells to non-tumor cells, including normal stromal, epithelial, and lymphatic cells. Genomic DNA was extracted using the Maxwell^®^ RSC Xcelerate DNA FFPE Kit (Promega, Mannheim, Germany).

The DNA isolation procedure using the Maxwell^®^ RSC Xcelerate DNA FFPE Kit (Promega) is characterized by a high degree of automation, which significantly enhances both its efficiency and reproducibility. Following the initial sample preparation, including dewaxing and protease digestion, subsequent extraction steps are carried out in a fully automated manner on the Maxwell^®^ RSC instrument. Samples are placed in single-use cartridges preloaded with the appropriate reagents, and running the dedicated program allows the isolation to be completed within approximately 30–60 min for up to 48 samples simultaneously. This technology considerably reduces manual handling time (hands-on time), while the resulting DNA isolates demonstrate high reproducibility and a lower risk of contamination compared to manual methods based on silica columns. Automated DNA isolation using the Maxwell^®^ RSC Xcelerate DNA FFPE Kit therefore represents a fast, intuitive, and efficient solution, particularly suitable for diagnostic and research laboratories where both high-quality nucleic acids and standardized analytical processes are essential.

### 2.2. DNA Quantification and Degradation Index

To assess the quality and quantity of DNA in the FFPE biological samples, the Quantifiler Trio DNA Quantification Kit (Thermo Fisher Scientific, Waltham, MA, USA) was used, following the manufacturer’s protocol. This kit amplifies three target sequences: short fragments (80 bp)—total human DNA quantification, long fragments (214 bp)—DNA integrity assessment, and a sex marker (Y chromosome, 75 bp)—detection of male DNA. The Quantifiler Trio method is critical for the analysis of low-quality samples, such as those commonly encountered in forensic investigations, as it enables the optimal selection of subsequent analytical methods, including PCR or NGS.

The Degradation Index (DI) was calculated as the ratio of the quantity of long-fragment DNA to short-fragment DNA. A DI value > 1 indicates DNA fragmentation—the higher the value, the greater the degree of sample degradation.

### 2.3. STR Amplification

For amplification of Short Tandem Repeat (STR) markers, the GlobalFiler™ PCR Amplification Kit (Thermo Fisher Scientific, Waltham, MA, USA) was employed. This multiplex PCR kit allows for the simultaneous amplification of 24 STR markers and the sex-determining marker (amelogenin). Amplification was carried out according to the manufacturer’s protocol. The GlobalFiler™ system enables the generation of DNA profiles even from low-quality and degraded samples, such as FFPE tissues. The method is characterized by high sensitivity, allowing amplification from as little as 0.1 ng of input DNA, while maintaining strong resistance to PCR inhibition.

### 2.4. Detection of PCR Products

Following amplification, the PCR products were separated by capillary electrophoresis on an Applied Biosystems 3500 Genetic Analyzer. Genetic profiles were analyzed and interpreted using GeneMapper™ ID-X Software v1.6 (Applied Biosystems, Foster City, CA, USA) with an analytical threshold of 100 RFU. Quality control measures included the evaluation of stutter peaks according to kit-specific thresholds GlobalFiler, (Thermo Fisher Scientific, Waltham, MA, USA), assessment of allele balance (heterozygote ratio 60:40), and identification of allele dropout. Negative controls were included in each run to monitor contamination.

## 3. Results

**Figure 1:**
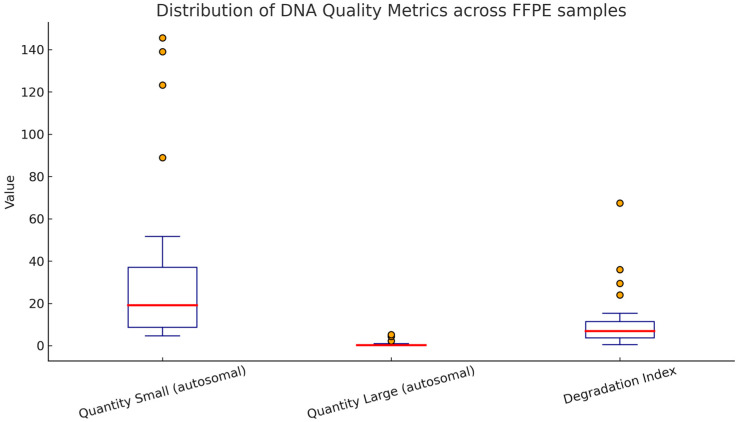
Distribution of degradation index and autosomal DNA quantities in FFPE samples.

The chart (Figure 1) presents three key DNA quality parameters from FFPE samples:

Quantity Small (autosomal)—Values are relatively high, indicating that short DNA fragments amplify much better and are better preserved.

Quantity Large (autosomal)—Values are very low and scattered, confirming that long DNA fragments undergo degradation and have limited usefulness in STR analyses.

Degradation Index—the distribution is broad, with several outliers (>100), showing substantial variability in DNA quality among samples; a high DI indicates severe DNA degradation.

DNA from FFPE blocks is characterized by better amplification of short fragments, whereas longer ones are significantly degraded, which is reflected in high DI values.

### 3.1. Legend

IPC (Internal Positive Control): Ct values of ~27–28 are stable, indicating the absence of strong PCR inhibitors. This suggests that the issue is not inhibition but rather DNA degradation.

Large autosomal amplicon (~300 bp): very low DNA quantities (often <1 ng/µL).

Small autosomal amplicon (~80–100 bp): significantly higher DNA quantities in most samples.

Degradation Index (DI): calculated as the ratio of DNA quantity from the small amplicon to the large amplicon.

DI ~ 1–3 → DNA moderately preserved, full STR profiles are possible.

DI > 10 → DNA strongly degraded, only partial STR profiles expected.

DI > 50–100 → extreme degradation, requiring miniSTR or NGS target enrichment.

Samples with a low degradation index (DI 4–7) (Table 1) contained relatively well-preserved DNA, which makes it possible to obtain full or nearly full STR profiles, provided that the input material is sufficient.

Samples with a medium DI (15–40) (Table 1) indicated moderate DNA degradation. In such cases, there is a high probability of obtaining only partial STR profiles. Nonetheless, analysis is feasible, particularly when using markers with shorter amplicon lengths (miniSTRs) or alternatively SNP panels.

In contrast, samples with a very high DI (≥80) (Table 1) exhibited severely degraded DNA. In these situations, conventional STR profiling usually fails or yields only trace results with a high risk of allele dropout. Therefore, advanced approaches such as miniSTRs, next-generation sequencing (NGS), or mitochondrial DNA analysis are required.

The results of DNA quantification from FFPE tissues demonstrated substantial quality limitations of the extracted genetic material. Elevated Degradation Index (DI) values, in some cases exceeding 100, clearly indicated a predominance of short DNA fragments accompanied by a pronounced scarcity of longer amplicons. These characteristics are typical for formalin-fixed, paraffin-embedded samples, in which DNA–protein cross-linking and fragmentation processes significantly compromise genomic integrity. Consequently, despite relatively high concentrations of short autosomal fragments, amplification of STR markers using the Global Filer kit was restricted to a limited number of loci with the shortest amplicons. The resulting STR profiles were therefore only partial and exhibited low evidential value. These findings suggest that in cases of severely degraded FFPE-derived DNA, alternative approaches such as mini-STRs, SNP panels, or mitochondrial DNA analysis, which rely on shorter amplicons, may provide more reliable and informative genetic data.

### 3.2. Forensic Relevance

In the case of samples with a low degradation index (DI), it is possible to obtain complete STR profiles that are fully compatible with identification systems such as CODIS and ESS [27,28,29]. Such profiles provide high discriminatory power and enable unambiguous personal identification as well as reliable database comparisons.

However, in severely degraded samples, conventional STR typing often fails due to allele dropout, preferential amplification of shorter loci, and reduced overall information content. In such cases, the application of modern SNP panels analyzed by next-generation sequencing (NGS) becomes essential. SNP markers offer significant advantages in the analysis of degraded DNA because they can be targeted with very short amplicons (60–100 bp), increasing the likelihood of successful amplification from fragmented material [30,31,32]. Moreover, SNPs provide additional utility in kinship testing and ancestry inference, expanding their forensic value beyond classical STR-based identification.

NGS further enhances this approach by allowing the simultaneous multiplexed analysis of hundreds to thousands of SNPs, combined with bioinformatic algorithms that correct for damage-induced artifacts typical of FFPE and other archival samples. This makes SNP/NGS-based analysis a powerful complementary strategy in forensic casework, especially when working with archival histopathological blocks or highly degraded biological material [33,34,35,36].

**Figure 2:**
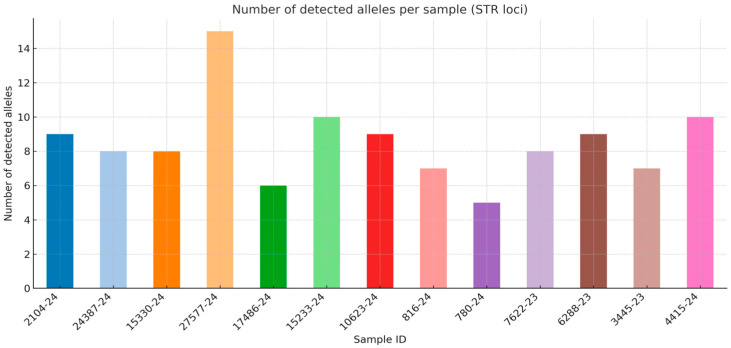
Number of successfully amplified STR alleles per FFPE sample.

The number of detected STR alleles was compared across FFPE-derived DNA samples. The bar chart illustrates the allelic recovery per sample, highlighting differences in amplification success and the variability in STR profile completeness (Figure 2).

The STR profiles obtained from the analyzed FFPE samples (Table 2 and Table 3) exhibited considerable variability in terms of completeness and amplification quality.

Analysis of the STR profiles derived from FFPE material revealed substantial heterogeneity in both completeness and data quality. In some cases, relatively rich profiles were obtained, encompassing a dozen or more markers (e.g., 2104-24, 27577-24, 6288-23, 4415-24), which may be applicable in both individual identification and kinship analysis. However, in certain samples, the presence of more than two alleles in a single locus was observed (e.g., D8S1179 and D5S818 in sample 27577-24, FGA in sample 4415-24), which may reflect formalin-induced artifacts or indicate potential DNA mixtures.

A considerable number of samples yielded only partial profiles, with extensive allele dropout and single alleles present in heterozygous loci. Examples include samples in which the number of identified markers was very limited (e.g., 17486-24, 15233-24, 780-24, 3445-23). Such results considerably limit their usefulness for individual identification, but they may still provide supportive information in genealogical and kinship analyses.

In summary, STR profiles obtained from FFPE samples were, in most cases, fragmentary and burdened with interpretational uncertainty due to allele dropout and the occurrence of additional alleles. Only a few samples allowed for the generation of profiles with potential individual identification value. In forensic practice, such results are primarily applicable in kinship analyses. Full exploitation of FFPE-derived DNA requires alternative approaches, such as miniSTRs, SNP panels analyzed by next-generation sequencing (NGS), or mitochondrial DNA analysis, which are better suited for highly degraded genetic material.

For the remaining samples, no amplification of STR markers was obtained, which is fully consistent with the DNA quantification results. The elevated Degradation Index (DI) values indicated the presence of only short DNA fragments, with a near-complete absence of longer sequences required for amplification of STR loci. Consequently, despite PCR being performed under standard conditions, no detectable signals corresponding to genetic markers were observed, reflecting the insufficient quantity of intact DNA capable of successful amplification. These findings confirm that in cases of severely degraded FFPE-derived material, conventional STR systems may yield no informative results, and alternative approaches targeting shorter amplicons, such as mini-STRs, SNP panels, or mitochondrial DNA analysis, should be considered.

From a forensic perspective, STR profiles obtained from FFPE tissues carry limited value for direct individual identification. Complete profiles, suitable for database comparisons in CODIS or ESS, were achieved only in rare cases. More commonly, partial profiles were recorded, prone to interpretational errors associated with allele dropout, additional alleles, and low signal quality. Nevertheless, even fragmentary results may represent a valuable source of information in kinship analysis, especially when compared with profiles obtained from related individuals.

As mentioned above, FFPE material demonstrates significant limitations in conventional STR profiling, and its usefulness for individual identification remains restricted. Nevertheless, in forensic practice, such samples may still provide value in genealogical and kinship investigations, particularly when complemented by analytical strategies specifically designed for highly degraded DNA. Approaches employing reduced amplicon size or alternative genetic targets can mitigate the effects of fragmentation, thereby enhancing the reliability of the obtained results and supporting the interpretation of data derived from formalin-fixed tissues.

## 4. Discussion

DNA profiling from FFPE samples is associated with numerous challenges, including DNA degradation, low extraction efficiency, and potential sequencing artifacts. Nevertheless, the development of new technologies and analytical approaches, including the application of artificial intelligence and alternative techniques, opens up new possibilities for improving the quality and reliability of results obtained from this type of material.

Analysis of Short Tandem Repeats (STRs) from FFPE (formalin-fixed, paraffin-embedded) samples represents an important tool in forensic genetics [19], molecular diagnostics [37,38], retrospective studies [39], and personalized therapy selection [40]. Nevertheless, this type of biological material poses a number of analytical challenges, which are also evident in the obtained results.

In the analyzed profiles, frequent data loss was observed in certain loci (e.g., CSF1PO, TPOX, DYS391, D2S1338, SE33), reflecting DNA fragmentation and allelic dropout. This phenomenon, typical for degraded DNA, may lead to misinterpretation of heterozygotes as homozygotes. Irregular amplification in loci such as SE33 or FGA further highlights the challenges of working with FFPE-derived DNA. Despite these limitations, most samples yielded 10–12 informative loci, which remains sufficient for comparative and identification purposes, although optimization strategies (e.g., miniSTRs, repeated amplifications) are required to improve reliability.

Many researchers emphasize the problem of DNA degradation in FFPE samples. Comparative studies have shown that FFPE-derived DNA is of lower quality and shorter fragment length than cryopreserved samples, with significantly reduced yields [3,36]. Moreover, fixation-induced artifacts, such as cytosine deamination to thymine, can introduce sequencing errors and compromise data accuracy [22,41]. To address these challenges, novel approaches, including AI-assisted methods such as the SmartPath system, have been proposed to optimize tissue selection for macrodissection, thereby improving DNA recovery and sample purity [42].

The obtained results highlight the need for particular caution when interpreting STR profiles that include fewer than 15 loci. The literature indicates that as the number of recovered markers decreases, the discriminatory power of the profile is significantly reduced, while the risk of misinterpretation increases due to phenomena such as allele dropout, drop-in, or artifacts associated with DNA degradation [43]. Partial profiles, especially from degraded material, may still contain useful information; however, they require complementary statistical analysis, such as calculating the Likelihood Ratio (LR) or the Combined Probability of Inclusion/Exclusion (CPI/CPE), which enables an objective assessment of the evidential value of the obtained data [44,45].

It should also be emphasized that despite numerous technical limitations and the risk of artifacts, FFPE tissues may represent the only available source of DNA in a range of forensic contexts. This is particularly relevant in cases of human identification, where archival histopathological samples may be the only preserved material [1]. In inheritance disputes, archival paraffin blocks can provide the sole means of obtaining the DNA profile of a deceased person in order to resolve questions of succession. Likewise, in cases of missing persons or suspected medical malpractice, FFPE material serves as a valuable source of information, enabling retrospective genetic analysis. Publications addressing the use of paraffin-embedded samples in forensic investigations clearly indicate that, despite the technical challenges associated with DNA degradation, this approach holds significant practical value and is increasingly supplemented with miniSTR, SNP, and next-generation sequencing (NGS) analyses. These complementary approaches allow for improved recovery of genetic information from highly degraded material, enhance the discriminatory power of partial profiles, and provide additional layers of evidence that can strengthen forensic and clinical interpretations, particularly in retrospective or otherwise challenging cases [4,8,35,46].

Despite its limitations, the forensic significance of FFPE tissues remains considerable—particularly in cases where no other sources of DNA are available. Modern approaches, such as miniSTR, qPCR, and Next Generation Sequencing, together with advances in bioinformatics, substantially improve the analysis of FFPE-derived DNA. These methods help to overcome the limitations associated with degradation and fragmentation, thereby increasing the reliability and evidential value of the results in forensic and clinical contexts.

Our study also demonstrates the practical advantages of the Maxwell^®^ RSC Xcelerate DNA FFPE Kit. The system is automated, user-friendly, and time-efficient, enabling the simultaneous extraction of up to 48 samples. These features make it attractive for forensic and clinical laboratories that process larger sample sets, offering reproducibility and reduced risk of contamination compared to manual protocols.

In conclusion, while DNA from FFPE material remains technically challenging due to degradation and fixation artifacts, its forensic and clinical relevance is considerable—particularly when no other sources of DNA are available. The integration of modern approaches, including miniSTRs, qPCR, NGS, and bioinformatics pipelines, significantly improves the reliability and evidential value of FFPE-derived profiles. The tested automated extraction kit further enhances workflow efficiency, supporting its potential role in routine applications.

## 5. Conclusions

1. FFPE tissues can be effectively utilized in STR profiling; however, this requires a careful approach for result validation and protocol optimization. Critical steps include the use of optimized DNA extraction methods, quality assessment of DNA prior to PCR, and cautious interpretation of results due to potential dropout and artifact occurrence.
2. The utility of FFPE samples depends primarily on the degradation index (DI), the number of amplified loci, and DNA concentration. Samples with DI < 10 and a concentration > 5 ng/μL can be used for full STR analysis, whereas for samples with DI > 50, alternative approaches such as SNP analysis, miniSTR, or mtDNA analysis should be considered.
3. DNA profiling from FFPE tissues in forensic practice carries inherent risks due to DNA degradation, but it can still provide valuable information when no other sources are available.
4. Our study indicates that the Maxwell^®^ RSC Xcelerate DNA FFPE Kit provides a convenient and efficient tool for DNA isolation from formalin-fixed, paraffin-embedded tissues. The automation of the process, the capacity to isolate up to 48 samples simultaneously, and high reproducibility make it an attractive solution for forensic and clinical laboratories, even though the obtained STR profiles are often partial and require careful interpretation.

## Figures and Tables

**Table 1:** Assessment of DNA concentration and Degradation Index (DI).

No.	Sample Name	Target Name	C_T_ Mean	Quantity Mean	Degradation Index
1	27338-23	T.IPC	27.28462982	-	-
	27338-23	T.Large Autosomal	28.03167725	0.191929743	51.59603119
	27338-23	T.Small Autosomal	23.33320999	9.902812958	51.59603119
2	780-24	T.IPC	27.39422417	-	-
	780-24	T.Large Autosomal	28.05417442	0.189387381	19.14279175
	780-24	T.Small Autosomal	24.87759399	3.625403166	19.14279175
3	24387-24	T.IPC	27.86660004	-	-
	24387-24	T.Large Autosomal	22.4848175	5.140621662	4.666538239
	24387-24	T.Small Autosomal	21.97338104	23.98890686	4.666538239
4	7622-23	T.IPC	27.42311668	-	-
	7622-23	T.Large Autosomal	26.39240265	0.507136106	18.29960823
	7622-23	T.Small Autosomal	23.43297958	9.280391693	18.29960823
5	47239-23	T.IPC	27.35767174	-	-
	47239-23	T.Large Autosomal	30.1026268	0.056239072	88.9747467
	47239-23	T.Small Autosomal	24.38232994	5.003857136	88.9747467
6	15330-24	T.IPC	27.6943512	-	-
	15330-24	T.Large Autosomal	25.44915199	0.887025833	5.82336092
	15330-24	T.Small Autosomal	24.33347511	5.165471554	5.82336092
7	27577-24	T.IPC	27.62685204	-	-
	27577-24	T.Large Autosomal	26.77074432	0.405258179	16.854599
	27577-24	T.Small Autosomal	23.90406418	6.830463886	16.854599
8	52934-23	T.IPC	27.72996712	-	-
	52934-23	T.Large Autosomal	31.37101936	0.026517242	23.76406479
	52934-23	T.Small Autosomal	27.56682968	0.630157471	23.76406479
9	816-24	T.IPC	27.65239334	-	-
	816-24	T.Large Autosomal	28.73755455	0.126309276	8.61333847
	816-24	T.Small Autosomal	26.72755241	1.087944508	8.61333847
10	2104-24	T.IPC	27.51393509	-	-
	2104-24	T.Large Autosomal	26.5255928	0.468639314	5.778486252
	2104-24	T.Small Autosomal	25.32598495	2.708025932	5.778486252
11	31954-23	T.IPC	27.62247467	-	-
	31954-23	T.Large Autosomal	27.56675911	0.252825886	36.40428925
	31954-23	T.Small Autosomal	23.44569206	9.203947067	36.40428925
12	3445-23	T.IPC	27.84559059	-	-
	3445-23	T.Large Autosomal	23.90898895	2.21006608	5.168225765
	3445-23	T.Small Autosomal	23.1138401	11.42212009	5.168225765
13	17486-24	T.IPC	28.19722366	-	-
	17486-24	T.Large Autosomal	31.04796028	0.032113694	15.91148281
	17486-24	T.Small Autosomal	27.88903999	0.510976493	15.91148281
14	18670-23	T.IPC	27.39790535	-	-
	18670-23	T.Large Autosomal	26.94467926	0.365558952	41.77180481
	18670-23	T.Small Autosomal	22.66760635	15.27005672	41.77180481
15	6180-24	T.IPC	27.63027191	-	-
	6180-24	T.Large Autosomal	31.10833549	0.030984785	145.5412598
	6180-24	T.Small Autosomal	24.54218292	4.509564877	145.5412598
16	1834-24	T.IPC	27.69294167	-	-
	1834-24	T.Large Autosomal	29.10348129	0.101680651	139.039566
	1834-24	T.Small Autosomal	22.78603172	14.13763428	139.039566
17	1754-23	T.IPC	28.40914917	-	-
	1754-23	T.Large Autosomal	30.97937202	0.033446159	36.38529587
	1754-23	T.Small Autosomal	26.55533028	1.21694839	36.38529587
18	11857-24	T.IPC	27.78021049	-	-
	11857-24	T.Large Autosomal	27.37384796	0.283452809	34.22826004
	11857-24	T.Small Autosomal	23.36468124	9.702096939	34.22826004
19	43248-24	T.IPC	28.07854462	-	-
	43248-24	T.Large Autosomal	29.62934685	0.074450895	37.04236221
	43248-24	T.Small Autosomal	25.29797173	2.757837057	37.04236221
20	6288-23	T.IPC	27.88058853	-	-
	6288-23	T.Large Autosomal	22.76756859	4.347402096	6.776962757
	6288-23	T.Small Autosomal	21.65751839	29.462183	6.776962757
21	5700-24	T.IPC	27.52523613	-	-
	5700-24	T.Large Autosomal	29.39518356	0.085535869	123.2788162
	5700-24	T.Small Autosomal	23.23667526	10.5447607	123.2788162
22	10623-24	T.IPC	27.41611862	-	-
	10623-24	T.Large Autosomal	26.42856407	0.496381819	13.13532639
	10623-24	T.Small Autosomal	23.97552681	6.52013731	13.13532639
23	55986-23	T.IPC	27.56273079	-	-
	55986-23	T.Large Autosomal	27.26762962	0.301872462	20.58268166
	55986-23	T.Small Autosomal	24.0496006	6.213344574	20.58268166
24	4415-24	T.IPC	27.99567986	-	-
	4415-24	T.Large Autosomal	22.76561165	4.352447987	8.263043404
	4415-24	T.Small Autosomal	21.35101891	35.96446609	8.263043404
25	15233-24	T.IPC	28.12033653	-	-
	15233-24	T.Large Autosomal	22.47094536	5.183064461	13.0022049
	15233-24	T.Small Autosomal	20.38585472	67.39126587	13.0022049

**Table 2:** Results of amplification using the GlobalFiler individual identification system (Applied Biosystems).

Locus/Sample	2104-24	24387-24	15330-24	27577-24	17486-24	15233-24	10623-24
D3S1358	15,16	14,16	16,18	16,17	15,16	16,17	14,16
vWA	14,15	15,17	14,16	15,16	15,17	17,18	14,18
D16S539	13	-	-	12,13	-	13	-
CSF1PO	-	-	-	12,13	-	-	-
TPOX	-	-	-	8	-	12	-
INS/DEL	-	-	-	-	-	-	-
AMELO	XX	XX	XX	XX	XX	XX	XX
D8S1179	14,16	12,13	14,15	12,13,14,15	12,13	11,16	10,13
D21S11	30,30.2	29,31.2	28,29	31.2,33.2	-	30	32.2
D18S51	15	16	19	13,14	-	14,15	-
DYS391	-	-	-	-	-	-	-
D2S441	10,14	10,11	11,14	11,13,14	11,14	14	10,14
D19S433	14,15.2	13,14	15	12,13	12,15	12,15	14,16
TH01	9.3	9.3	-	9.3	-	9,9.3	9,9.3
FGA	21,25	19	22	22,23	22	22	21
D22S1045	14,16	12,15	16	12,15	16	11	15
D5S818	9,10	10,12	11,12	9,11,12,13	12,13	12,13	12,13
D13S317	-	11	9,10	9,11	-	8,10	-
D7S820	8	9,10	9,11	7.3,10	-	-	10
SE33	-	24.2	-	14,20	-	-	-
D10S1248	14,16	15,17	13,16	13,17	12,13	14	14,15
D1S1656	13,14	12,14	12,17.3	-	16	15,17.3	16,17.3
D12S391	-	16,22	-	22	21	17.3,21	23
D2S1338	-	-	-	19	-	16	-
Locus/Sample	816-24	780-24	7622-23	6288-23	3445-23	4415-24	
D3S1358	14	13,16	15	15,18	15,17	16	
vWA	17	17	15,18	15,18	14,16	16,17	
D16S539	-	-	13	11	-	12	
CSF1PO	11	-	-	-	-	10,11	
TPOX	-	-	-	-	-	-	
INS/DEL	-	-	-	-	-	-	
AMELO	XX	XX	XX	XX	XX	XX	
D8S1179	10,12	10,14	10,13	13,14	10,14	10,14	
D21S11	29,32.2	-	30,32.2	29,30	33.2	29,31.2	
D18S51	16,17	-	-	13,15	16	-	
DYS391	-	-	-	-	-	-	
D2S441	10,11	10,14	11.3,14	10,11	10,11	10,11	
D19S433	13,15	14,15	15,16.2	12,16.2	14.2,15	14,15	
TH01	7,9.3	6	9.3	9	9.3	7,9	
FGA	22	23	23,24	20,21	24	21,22,23	
D22S1045	14,16	16	11,12	14,17	11,15	16,17	
D5S818	11,13	11,12	10,12	12,13	9,12	11,12	
D13S317	10,13	8,10	-	9,13	8,10	8,10	
D7S820	-	10	9	8,11	11	8,12	
SE33	-	-	-	-	-	29.2,30.2	
D10S1248	14,17	13,14	13,16	13,16	13,14	14,15	
D1S1656	18.3	14,16	16.3,17.3	11,17	13,15	14,16.3	
D12S391	-	-	20	18	17	-	
D2S1338	-	-	-	16	-	-	

**Table 3:** Summary of STR Profile Completeness—FFPE Samples.

Sample Name	Number of STR Loci	Profile Status	Recommendation
2104-24	18	Complete profile	Suitable for STR-based identification
24387-24	19	Complete profile	Suitable for STR-based identification
15330-24	17	Complete profile	Suitable for STR-based identification
27577-24	18	Complete profile	Suitable for STR-based identification
17486-24	16	Complete profile	Suitable for STR-based identification
15233-24	14	Partial profile	Potential identification, validation recommended
10623-24	17	Complete profile	Suitable for STR-based identification
816-24	10	Partial profile	Useful for exclusion or SNP comparison
780-24	9	Partial profile	Suitable for SNP or mtDNA analysis
7622-23	11	Partial profile	Applicable for kinship comparisons
3445-23	7	Fragmented profile	Re-isolation/miniSTR suggested
4415-24	6	Fragmented profile	Incomplete, mtDNA may be considered
6288-23	7	Fragmented profile	Unusable as is, consider SNP/mtDNA

## Data Availability

The detailed data and electropherograms supporting the findings of this study are available from the corresponding author upon reasonable request (email: dagmara.lisman@pum.edu.pl).
